# Extracellular vesicles in the retina - putative roles in physiology and disease

**DOI:** 10.3389/fnmol.2022.1042469

**Published:** 2023-01-12

**Authors:** Aikaterini A. Kalargyrou, Siobhan E. Guilfoyle, Alexander J. Smith, Robin R. Ali, Rachael A. Pearson

**Affiliations:** ^1^King’s College London, Guy’s Hospital, Centre for Gene Therapy and Regenerative Medicine, London, United Kingdom; ^2^Kellogg Eye Center, University of Michigan, Ann Arbor, MI, United States

**Keywords:** extracellular vesicle, exosomes, microvesicles, retina, neuroprotection, cell therapies, degeneration, ocular therapies

## Abstract

The retina encompasses a network of neurons, glia and epithelial and vascular endothelia cells, all coordinating visual function. Traditionally, molecular information exchange in this tissue was thought to be orchestrated by synapses and gap junctions. Recent findings have revealed that many cell types are able to package and share molecular information *via* extracellular vesicles (EVs) and the technological advancements in visualisation and tracking of these delicate nanostructures has shown that the role of EVs in cell communication is pleiotropic. EVs are released under physiological conditions by many cells but they are also released during various disease stages, potentially reflecting the health status of the cells in their cargo. Little is known about the physiological role of EV release in the retina. However, administration of exogenous EVs *in vivo* after injury suggest a neurotrophic role, whilst photoreceptor transplantation in early stages of retina degeneration, EVs may facilitate interactions between photoreceptors and Müller glia cells. In this review, we consider some of the proposed roles for EVs in retinal physiology and discuss current evidence regarding their potential impact on ocular therapies *via* gene or cell replacement strategies and direct intraocular administration in the diseased eye.

## Introduction

### Cell communication in the retinal tissue

**Figure 1:**
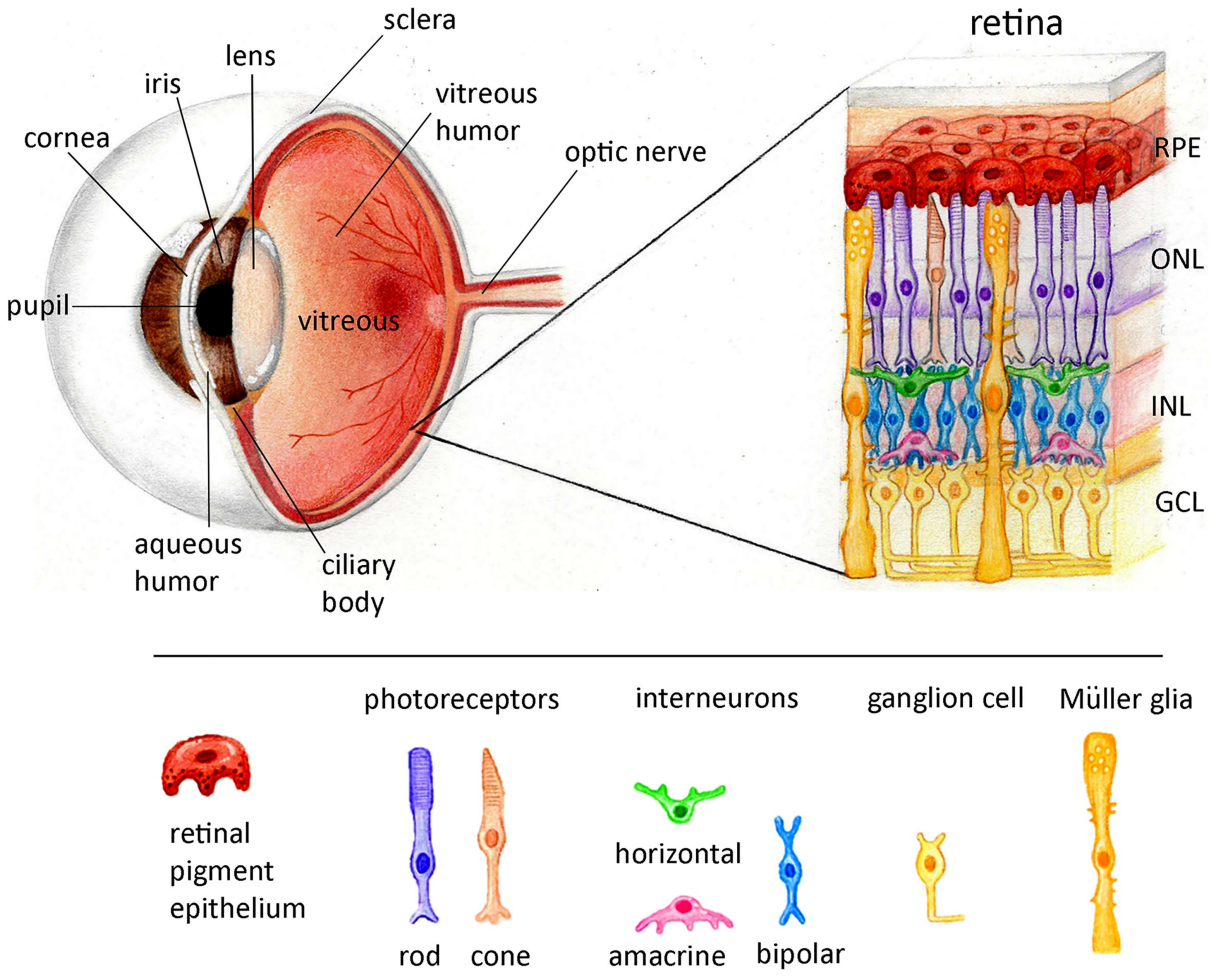
Schematic representation of the anatomical features of the eye and the lamination of the retina. The eye is anatomically divided in three layers. The external layer of the eye consists of the cornea, and sclera. The intermediate layer includes-at the anterior-the iris and ciliary body and in the posterior the choroid (not shown here). The internal layer is comprised by the retina. The space is filled with fluid called the aqueous humor, at the anterior chamber, or vitreous humor between the lens and the retina. The retina is divided in two compartments: one non-neuronal, the retina pigment epithelium (RPE) and one neuronal, the neuroretina. The vertebrate neuroretina is laminated in three layers of nerve cell bodies and two layers of synapses. The outer nuclear layer (ONL) consists of the somata of the rods and cones, the inner nuclear layer (INL) consists of the cell bodies of the bipolar, horizontal and amacrine interneurons and the ganglion cell layer (GCL) contains cell bodies of ganglion cells and some displaced amacrine cells. The axons of the ganglion cells are forming the optic nerve interconnecting the retina with the brain.

The retina is central for visual function and comprises several types of cells in a precise laminated orientation of two main structures, the non-neuronal retinal pigment epithelium (RPE) and the neuroretina (Figure 1). The vertebrate neuroretina is further organised into three layers of nerve cell bodies and two layers of synapses. Vision begins when photopigments called opsins absorb and become excited by light (reviewed in Yau and Hardie, 2009). Phototransduction is a tightly orchestrated process, starting in the outer segment, a highly specialised cilia, where the electrochemical signal is amplified and transmitted to an array of interneurons *via* synapses (rod spherules and cone pedicles) and gap junctions, before being transmitted to the brain *via* the retinal ganglion cells (Wald, 1951; VanHook, 2017). While the cells of the neuroretina routinely exchange information *via* gap junctions and synapses, they may also interact with each other *via* the release and uptake of extracellular vesicles (EVs). In this review we will discuss the role of EVs as intercellular carriers of information within the retina and their potential application in ocular therapies.

### Extracellular vesicular biogenesis: A brief overview of the endocytic machinery

EVs comprise a very diverse category of lipid-encapsulated carriers of molecular information released by cells (reviewed in Colombo et al., 2014; Tkach and Théry, 2016; van Niel et al., 2018a). Due to this being a relatively new field, terminology in the literature is mixed, but it is now commonly accepted that EVs are membrane-bound vesicles that may originate from the endocytic pathway [exosomes, 50-150 nm diameter (Johnstone et al., 1991)] or directly bleb from the plasma membrane (ectosomes, microvesicles, 30 nm-3 μm diameter, reviewed in Verweij et al., 2021). As such, they can contain a repertoire of proteins and genetic material as well as structural information from the cell cytosol, enclosed in a lipid bilayer.

Historically, the first description of EVs originating from mammalian cells dates back to 1987 where exosomes were referred to as by-products of reticulocyte maturation (Johnstone et al., 1991). However, recent research has shown that EVs contain both proteins and functional RNA species that can be taken up and translated by recipient cells (Valadi et al., 2007). The ability of EVs to exert regulatory activity on the genomes of target cells has led to EVs now being recognised as mediators of cellular communication and a potential agent in a plethora of disorders. Technological advancements in microscopy (Hyenne et al., 2019; Verweij et al., 2019; Bebelman et al., 2020; Mathieu et al., 2021), genetic engineering (Zomer et al., 2015; Verweij et al., 2018), flow cytometry (van der Vlist et al., 2012), and proteomic analysis (Kowal et al., 2016) have revealed the pleiotropic functions of the EV cargo, ranging from local cell–cell interactions within the same tissue to long range actions within an organism. However, the mechanistic details of preferential cytoplasmic material sorting in EVs, cell specific targeting and docking of EVs, and of how EV cargo avoids cytoplasmic degradation upon uptake are major questions that are yet to be answered.

EVs of endosomal origin correspond to membrane invaginations (inwards budding) of endosomes forming intralumenal vesicles (ILVs) that mature inside multivesicular bodies (MVBs) and may be referred to as late endosomes (reviewed in von Bartheld and Altick, 2011; Colombo et al., 2014). Some MVBs are fated for degradation, and fuse with lysosomes, whereas others can fuse with the plasma membrane, leading to the release of ILVs as exosomes (Colombo et al., 2013; Kowal et al., 2016). Sorting of the ILV cargo is a very complex process that is regulated by an array of proteins such as Endosomal Sorting Complexes Required for Transport (ESCRT) protein complexes (ESCRT-0, -I, -II and-III), Tetraspanins, Rab GTPases, calcium binding proteins such as ALIX and more. For example, siRNA knockdown of ESCRT protein groups and ALIX revealed that the EV cargo reflects the specific sorting mechanism (Colombo et al., 2013; Larios et al., 2020). Late endosomal compartments are often enriched in tetraspanins (TSPANs), such as CD63, that further support the regulation of cargo sorting (van Niel et al., 2011; Verweij et al., 2018; Mathieu et al., 2021). Rab GTPases promote trafficking between specific intracellular compartments (Ostrowski et al., 2010). Rab conversion, the replacement of Rab5 with Rab7, is a sign of progression from an early MVB to a mature MVB (Deinhardt et al., 2006), whereas Rab11/11a-positive compartments can also be sites of exosome biogenesis, and this mechanism is regulated by glutamine depletion (Fan et al., 2020). As such, EV cargo can exhibit very high levels of heterogeneity due to different sorting mechanisms and the cellular microenvironment [for example nutrient availability (Fan et al., 2020)]. Cargo diversity increases further still if we consider that EVs not only originate from the endocytic machinery but may also bud from the plasma membrane; therefore different types of EVs can represent a snapshot of different compartments of the cell, carrying information about a cell’s molecular processes and potentially reflecting its health status. For example, cells release apoptotic bodies (AB), a subtype of EVs, when undergoing cell death (reviewed by Santavanond et al., 2021) and the membrane properties along with the cargo of AB-EVs can differ from EVs from healthy cells of the same type (Théry et al., 2001).

There are some excellent reviews on EV biogenesis and cargo characterisation from the pioneers of the field and readers should refer to Colombo et al. (2013, 2014), Raposo and Stoorvogel (2013), Rajendran et al. (2014), Kowal et al. (2016), Tkach and Théry (2016), van Niel et al. (2018b, 2021, 2022), Mathieu et al. (2021), Santavanond et al. (2021), Verweij et al. (2021) as this is beyond the scope of the current review. Regarding EV biogenesis in the retina, many studies have focused on the endocytic pathway in retinal pigment epithelium (RPE) cells, mostly because of their extraordinary phagocytic and material degradation capabilities. Therefore, MVBs in RPE cells have predominantly been studied for their role in degradation and their potential interplay with phagosomes and melanosomes (Wavre-Shapton et al., 2014).

**Figure 2:**
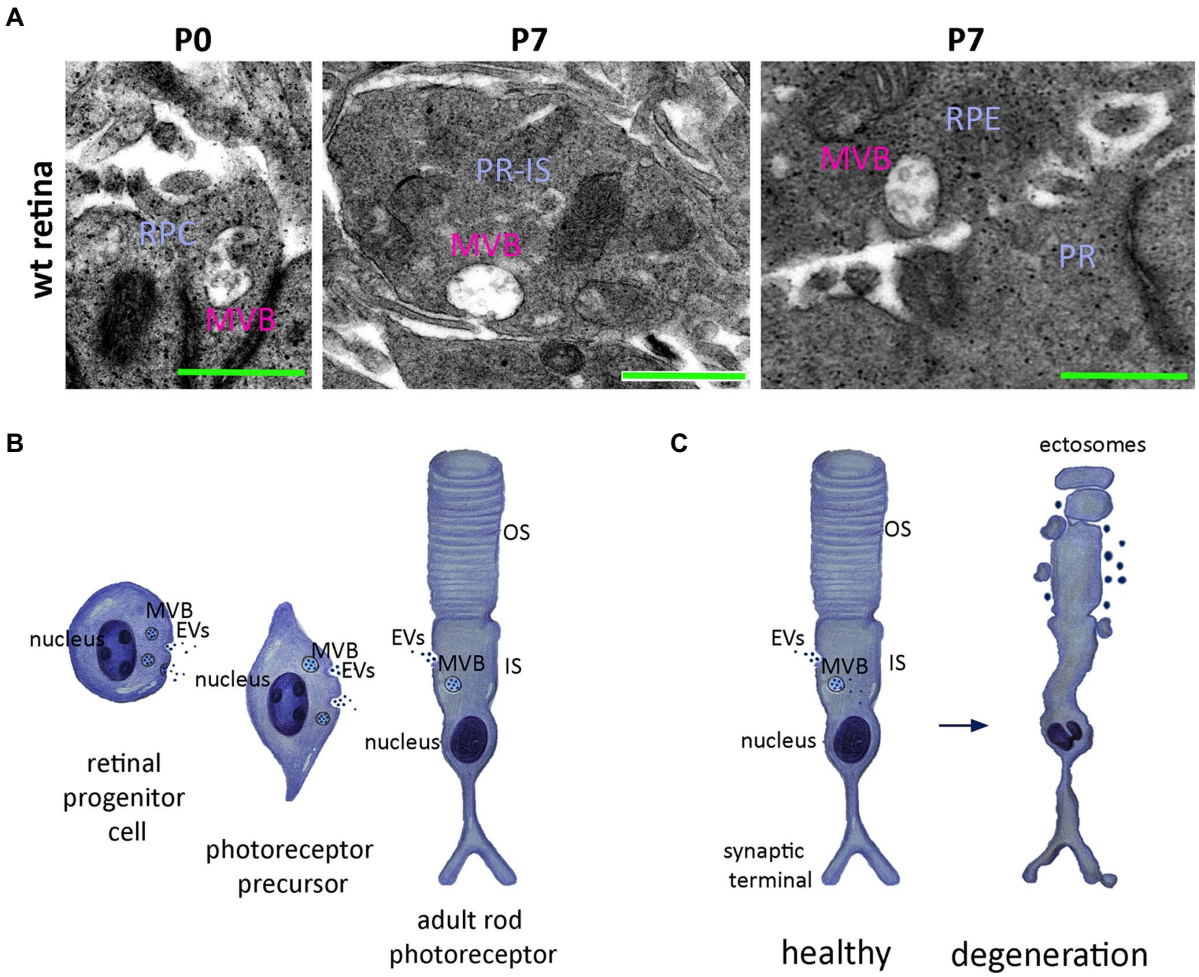
Potential roles of the extracellular vesicles release in mammalian rod photoreceptors. **(A)** Electro-microscopical analysis of the murine retina reveals Multivesicular bodies (MVBs) from left to right; in retinal progenitor cells (RPC) at postnatal day 0 (P0); at postnatal day 7 (P7) MVBs are obvious at the area of the developing photoreceptor inner segment (PR-IS), in close proximity to the photoreceptor membrane indicating potential release in the surrounding photoreceptors. On the right side, at P7 MVBs are also obvious in retinal pigmented epithelium (RPE) cell again in close proximity to the cell plasma membrane and in the apical side; facing the developing neuroretinal photoreceptors; **(B)** Schematic representation of the developing retinal progenitor cell (RPC) towards rod photoreceptor precursors indicating release of extracellular vesicles (EVs) originating either from MVBs or budding from the membrane. The release is reduced in adult stages of photoreceptor development where the cell has formed the inner (IS) and outer (OS) segment; (**C**) Schematic representation of the ectosome release by the malfunctioning rod photoreceptor cilium-the outer segment- in *rds^-/-^* mutant photoreceptors (right) compared to the normal MVB biogenesis and release or direct building of EVs in healthy photoreceptors.

The endocytic machinery of vertebrate photoreceptors has been studied extensively in amphibians, and most of our knowledge comes from following the endocytic pathway of gold labelled nanoparticles using electron microscopy. MVBs are localised in the inner segment or presynaptic terminals of frog photoreceptors (Hollyfield and Rayborn, 1987; Schmied and Holtzman, 1987; Sulzer and Holtzman, 1989). Their localisation in presynaptic terminals may indicate a role for MVB-regulated degradation and recycling in supporting synaptic transmission, as proposed for other neuronal cells (Hollyfield et al., 1985; Hollyfield and Rayborn, 1987; Schmied and Holtzman, 1987; Sulzer and Holtzman, 1989). Notably, elsewhere in the brain the presence of increased numbers of MVBs in neurons has been linked with cell dystrophy (Montiani-Ferreira et al., 2005; Altick et al., 2009). Although exocytosis events and EV release have been shown in frog photoreceptors (Hollyfield and Rayborn, 1987; Schmied and Holtzman, 1987; Sulzer and Holtzman, 1989), such evidence is missing for mammalian photoreceptor cells. Analysis of the mammalian photoreceptor ultrastructure also reveals MVBs localised in the soma and/or nascent inner segment of the early postnatal murine retina in subcellular areas of photoreceptor in close proximity to Müller glia cells (Kalargyrou et al., 2021; see also Figure 2A). The number and localisation of MVBs within photoreceptor cell inner segments may be indicative of a role in recycling and synthesis of the cell membrane, which is turned over at an astonishing rate compared to many other cell types (Calderón et al., 2002). Whether increases in MVB formation corresponds to increased release of EVs is as yet unknown.

## Retina cells can release extracellular vesicles during early development and in pathological conditions

EVs are released from all eukaryotic cell types; their secretion is evolutionarily conserved, and their roles are varied (Colombo et al., 2013; Raposo and Stoorvogel, 2013). For the purposes of this review, we will focus on the published literature pertaining to the release of EVs by mammalian retinal cells, either *in vivo*, *ex vivo* or in primary cultures. We consider some of the proposed role(s) of retinal EVs in normal and pathophysiological responses, together with how they are being explored as potential therapeutic agents.

### Retina progenitor cells release EVs during early stages of retina development

During retinal development, the different cell types originate from a common population of multipotent retinal progenitor cells (RPCs) (Turner and Cepko, 1987). Retinal development is orchestrated by a combination of intrinsic and extrinsic signalling pathways (Belliveau and Cepko, 1999; Cepko, 1999; Swaroop et al., 2010). Intrinsic signals refer to networks of transcription factors that tightly regulate the fate of cells, such as homeodomain proteins [see (Swaroop et al., 2010)]. However, cell specification is also influenced by extrinsic signals, soluble factors, in the extracellular environment (Belliveau and Cepko, 1999; Cepko, 1999). The discovery of EVs has led to a re-consideration of their potential role as delivery systems for some of these important signalling molecules. RPCs in culture have been shown to release EVs, and RNA profiling suggests that they may contain mRNA of transcription factors responsible for cell specification and multipotency, such as Pax6, Hes1, Ki-67, Sox2 and Nestin (Zhou et al., 2018). Similarly, Let7d, miR-9, miR-182 and miR-204 miRNA molecules with known roles in retinal development were found to be enriched in RPC derived EVs (Zhou et al., 2018). In addition, the same group reported finding mRNA species associated with mRNA transcription and transport, such as poly(A)-binding protein cytoplasmic 4 (Pabpc4), in RPC EVs (Zhou et al., 2018). This is note-worthy since these molecules function to support mRNA expression by increasing mRNA stability and protecting it from decay upon translation (Kini et al., 2014). Although pioneer studies on RNA EV cargo have shown that some mRNA molecules contained within can be translated by recipient cells (Valadi et al., 2007), debate remains around whether this means that only some or all mRNA species contained within EVs are translated into functional protein by recipient cells.

In addition to mRNA species, RPC-EVs also present with an array of proteins, but the majority are related to MVB sorting, vesicle assembly and transport, as well as integrin subunits (Zhou et al., 2018). In contrast to the mRNA profile, no proteins related to retinal development or function were identified, with the exception of Slc1a2, a glutamate transporter. Whilst this study provided a detailed characterisation of the content of RPC-EVs, evidence of functional uptake of these RPC-EVs by other RPCs and/or other retinal neurons is limited, and further work is required to ascertain the biological relevance of these RPC-EVs with regards to retinal development.

Recently, the same group reported the release of EVs from human retinal organoids, and performed an in-depth transcriptomic characterisation of the RNA content (miRNAs, piRNAs and tRNA) of these EVs at three different developmental timepoints, at day (D)42, D63 and D90 (Zhou et al., 2021). At D42 organoids predominantly comprise Pax6+ undifferentiated RPCs that are multipotent and proliferating; by D63 they contain fewer RPCs but many immature photoreceptor precursors and ganglion cells, and at D90 the majority of cells are differentiated with few RPCs. The miRNAs detected in organoid-derived EVs corresponded with a small subset of the miRNAs expressed by the cells (mixed population derived from the organoids) at each respective time point (Zhou et al., 2021). Between D42 and D90, the authors found a 10-fold reduction in total miRNA content within the EV preps collected and propose that RPCs are the major cell type contributing to EV release (Zhou et al., 2021). This raises potentially interesting questions as to the function of these RPC-derived EVs. Zhou and colleagues sought to explore whether RPC-derived EVs had any measurable effect on neighbouring RPCs: hRPCs were incubated with EVs derived from D42 whole organoids, *in vitro* (Zhou et al., 2021). The authors observed a downregulation of *CCSER2, PVRL1, FAM117B, ILDR2* and *CTDSPL* targeted genes (Zhou et al., 2021). However, the role of these genes in retinal development and thus the functional significance of these changes is currently unclear. Perhaps of more immediate relevance, the authors also observed an upregulation of *RLBP1* expression (Zhou et al., 2021). The *RLBP1* gene encodes cellular retinaldehyde binding protein that is responsible of the conversion of 11-trans-retinal to the light sensitive 11-cis retinal in Müller glia cells and RPE, supporting visual function (Sato et al., 2016). The time course of the observed RPC EV induced upregulation of *RLBP1* expression is consistent with Muller glial maturation in hPSC-derived retinal organoid cultures (Ning et al., 2022). However, more work is required to determine whether RPC-derived EVs play a direct role in signalling and specification of neighbouring RPCs towards a Muller glial fate. The reported presence of CDK11 and RGPD miRNA in EVs from retinal organoids(Zhou et al., 2021) is also interesting, given recent studies showing the potential for selective mechanisms of sorting of RNA species into EVs (Clancy et al., 2019); these are genes with known roles in facilitating cell proliferation (*CDK11*) and cell specification (*RGPD*) (Zhou et al., 2021). Whilst these transcriptomic analyses present a number of interesting observations, an important caveat is that both the EVs and cells were derived from whole organoids, which comprise a heterogeneous mix of cell types, not a single cell type, such as RPCs; as such, some caution is required when extrapolating the potential functional roles of these EV populations in retinal development, as represented in the schematic, Figure 2B.

### Vesicular release by retinal neurons may indicate cell dysfunction

Photoreceptors are the light sensing cells of the eye and constitute over 70% of all neural retinal cells, with rods and cones occurring at a ratio of 30:1 in mice and 20:1 in humans (Carter-Dawson and Lavail, 1979a, b). Both rod and cone photoreceptors are highly polarized neurons. They consist of an outer segment (OS), connecting cilium (CC), inner segment (IS), nucleus and synaptic terminal. The phototransduction proteins are organised in the OS, a non-motile primary cilium (for a review refer to Yau and Hardie, 2009), which in the rods has a cylindrical architecture comprised of up to 2,000 flattened membranous discs for efficient light capture (Sjostrand, 1953; Nickell et al., 2007).

Elsewhere, it has been shown that primary cilia are able to release a type of EV that originates from membrane blebbing and termed ciliary ectosomes (reviewed in Wood and Rosenbaum, 2015). In healthy photoreceptors, ectosome release is supressed by peripherin (Molday and Goldberg, 2017; Salinas et al., 2017), a tetraspanin protein responsible for membrane bending. Specifically, Ashavsky‘s group compared rod photoreceptors in the *Prph2^rd2/rd2^* mouse, a naturally occurring mouse line with a mutation in the peripherin gene, *Prph2*, against wildtype rods throughout development from early postnatal through to adult (Salinas et al., 2017). In *Prph2^rd2/rd2^* mice, the interphotoreceptor matrix (IPM) is increasingly filled with vesicular material, which had previously thought to be debris associated with photoreceptor degeneration (Cohen, 1983). In marked contrast, whilst a small amount of ectosomal release was observed at postnatal day (P)10 in wildtype retinas, none was seen after P14 (Salinas et al., 2017). In an elegant series of experiments, in which they overexpressed either the C-terminus of peripherin or the tetraspanin core fused with the rhodopsin C-terminus in retinal explants, Ashavsky‘s group showed that the C-terminus domain of peripherin is responsible for the suppression of ciliary ectosome production (summarised in Figure 2C), while the tetraspanin core induces membrane curvature (Salinas et al., 2017; Spencer et al., 2020).

Recently, our group showed that healthy photoreceptors do possess the machinery necessary to make and releasing EVs in culture, at least during the early postnatal (P0-P8) period of development (Kalargyrou et al., 2021), a finding also confirmed by Wallace and colleagues (Ortin-Martinez et al., 2021). During early postnatal development, photoreceptor derived EVs (PR-EVs) isolated from primary cultures, were found to be enriched with the early endocytic marker LAMP1, as well as phototransduction proteins including rhodopsin and recoverin and mRNA for rod a-transducin (Kalargyrou et al., 2021). We employed Cre-loxP mediated recombination to explore the possible uptake of PR-EVs *in vivo*, driving the expression of Cre recombinase under the control of the promoter for the rod-specific transcription factor, *Nrl* (Akimoto et al., 2006). PR-EVs were isolated from *Nrl.Cre^+/+^* rod photoreceptor primary cultures and injected into either the sub-retinal or intravitreal space of *TdTomato^fl/fl^* reporter mice. Surprisingly, no recombination was observed in RPE cells, even following subretinal administration of Nrl.Cre + EVs, potentially indicating some neuron to glia specificity (Kalargyrou et al., 2021). Regardless of injection site, we observed specific Cre-LoxP recombination in Müller glia cells with little or no TdTomato expression in other cell types, including photoreceptors (Kalargyrou et al., 2021). However, when we generated Nrl.Cre x TdTomato^fl/fl^ chimeric mice, no Müller glia cells expressed TdTomato (other cell types did) indicating that, in the healthy intact retina the release of EVs is not a key mediator of physiological functions in between neurons or neurons to glial cells (Kalargyrou et al., 2021). It remains possible, however, that EV release may be important in the degenerate retina or under conditions of cell stress, in line with other reports (Salinas et al., 2017; Spencer et al., 2020; Kalargyrou et al., 2021; Ortin-Martinez et al., 2021).

Explant cultures of retinas exhibiting photoreceptor degeneration (*Rd10*) do show an increase in EV release, compared with wildtype controls, supporting the idea that EV release is associated with photoreceptor pathology and/or stress (Vidal-Gil et al., 2019). However, it must be noted that in this study the EVs were isolated from whole retinal explants; while photoreceptors make up the majority of retinal cells, it is also very reasonable to assume that other cell types are contributing to EV release, such as Müller glia cells or interneurons, and that these cells might increase their EV release in response to retinal stress.

Proteomic profiling with Liquid Chromatography Mass Spectrometry (LC–MS) of EVs derived from adult wildtype retinal explants revealed that the majority of EV proteins were ESCRT related, indicating a potential origin from multivesicular bodies (MVBs) (Mighty et al., 2020). On the other hand, only very few proteins with predicted roles in retinal function, such as cadherin related family member (Cdhr1), castor zinc finger 1 (Casz1), syndecan-binding protein (Sdcbp), and retinol dehydrogenase 5 (Rdh5), were detected (Mighty et al., 2020). RT-QPCR analysis revealed mRNA transcripts for *Rhodopsin* and *NeuN*, along with *Gpcrc5b, Igsf8* and *Smoc-1* (Mighty et al., 2020). In contrast with *Rhodopsin*, *Gpcrc5b, Igsf8* and *Smoc-1* are not known to exert a specific retinal function. Since NeuN is an interneuron specific marker, it has been proposed that inner retinal neurons may also release EVs (Schlamp et al., 2013), although this has yet to be explored further.

Retinal ganglion cells (RGCs) are the connecting neurons to the brain and their axons forming the optic nerve. Several publications have suggested possible neuroprotective effects of EVs derived from other cell types on RGCs (discussed in section 4). However, to our knowledge, only one group has addressed the ability of rat RGCs themselves to release EVs in culture (Wang et al., 2021). The role of RGC-derived EVs is not yet known but when pre-labelled with lipophilic tracers and administered *in vivo via* intravitreal injection they were shown to adhere to the cell membrane of other RGCs (Wang et al., 2021). It is important to note that this does not necessarily confer specificity of uptake and may simply reflect anatomical proximity so further work *in vitro* with other cell types would help determine if RGC-EVs are indeed specifically taken up by other neighbouring RGCs and/or other cell types.

To summarise, while there is some evidence to indicate that retinal neurons are capable of release EVs *in vitro*, it remains to be shown if such release occurs in the intact adult mammalian eye under normal physiological conditions (Figures 2B,C).

### Pathology-induced changes in RPE derived EV cargo

**Figure 3:**
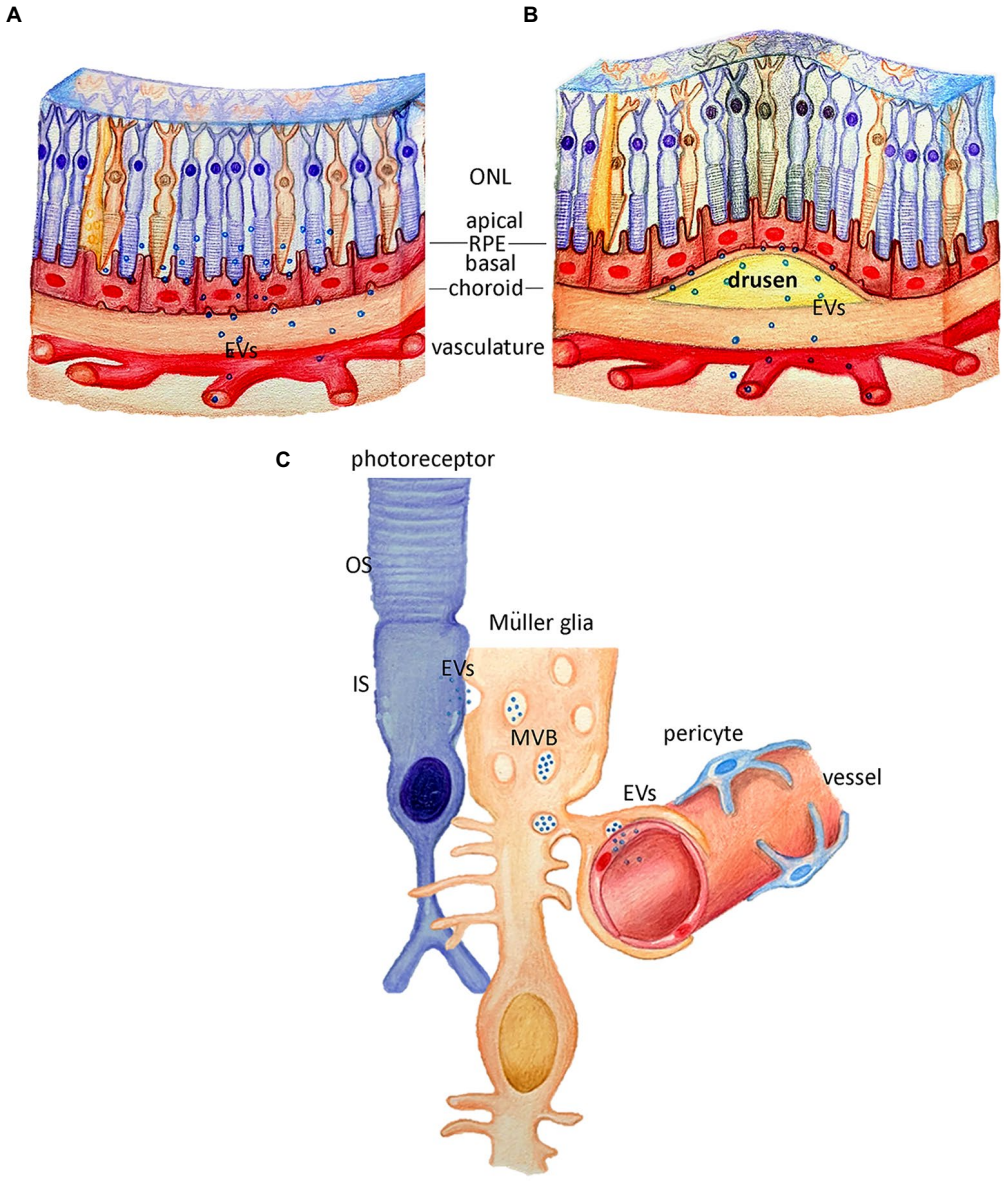
Potential roles of extracellular vesicles in retina pigment epithelium and Müller Glia cells. **(A)** Schematic representation of the retina, where retina pigment epithelium (RPE) is potentially releasing extracellular vesicles (EVs) from the apical and basal side under healthy conditions. **(B)** Schematic representation of the retina with drusen formation, where the RPE EV release from the basal side, may potentially contribute to the drusen pathology. **(C)** Schematic representation of the suggested roles of the EVs release by the Müller glia cells, either *via* MVB release of direct budding, under healthy conditions where the EVs can be released towards photoreceptors or towards the vascular endothelium.

The retina pigment epithelium (RPE) is a monolayer mainly responsible for the preservation and homeostasis of the outer neuroretina by actively phagocytosing the photoreceptor outer segments, supporting membrane turnover and phototransduction (Young, 1967; Lavail, 1973) (see Strauss, 2005). It also forms the outer blood-retina barrier; from the choroid-facing, basal side, it takes up nutrients such as glucose, retinol, and fatty acids from the blood to the neuroretina, and, from the retinal-facing, apical side, it transports ions, water, and metabolic end products from the neuroretina to the blood stream (Cunha-Vaz, 1976; Boulton and Dayhaw-Barker, 2001). Recently, it has been reported that RPE cells release different types of EVs from the apical and the basal compartment (Figure 3A; Locke et al., 2014; Klingeborn et al., 2017; Shah et al., 2018; Flores-Bellver et al., 2021). The potential roles of these EVs in RPE (dys)function are an area of recent interest.

Proteomic profiling of the EVs collected from human and porcine induced pluripotent stem cell (iPSC) derived RPE has shown a variety of extracellular matrix (ECM) and heat shock proteins encapsulated in RPE-derived EVs (Klingeborn et al., 2017). However, due to technical challenges, these results require further validation with immunolabeling as contaminants from outside EVs can co-precipitate due to the isolation process (Norman et al., 2021). Moreover, it is unclear if RPE derived from iPSCs produce and release EVs in the same way as primary RPE cells, so a degree of caution should be taken when extrapolating such findings to the normal intact RPE.

Electron microscopy (EM) with immunogold labelling revealed the presence of αB crystallin both inside and outside the lumen of RPE-derived EVs (Sreekumar et al., 2010). Specifically, it is enriched in the lumen of EVs derived from the apical, retinal facing side of human RPE in culture, whilst it is associated with the membrane of EVs produced by the basal side, at least under conditions of cell stress (Sreekumar et al., 2010), indicating differential cargo sorting in EVs based on cell polarity (Sreekumar et al., 2010; Klingeborn et al., 2017; Flores-Bellver et al., 2021). αB crystallin is a chaperone protein that plays an important role in mitochondria-induced cell stress, and is usually found in the cytosol and in the mitochondria (Yaung et al., 2007). However, it is accumulated in subretinal drusen deposits, a feature of age-related macular degeneration (AMD), and it is proposed that this accumulation may be partially due to altered EV transport (Sreekumar et al., 2010; Flores-Bellver et al., 2021).

Other candidate proteins known to be present in drusen were also found in RPE-derived EVs *via* proteomic analysis, including APOE, Aβ, VTN, VIN and CLU (Flores-Bellver et al., 2021). Like αB crystallin, these proteins are present in EVs, but direct (non-EV related) secretion cannot be excluded. The mechanism of EV release from RPE cells is yet to be fully elucidated but G-protein coupled receptors are reported to be able to regulate exosome release (Verweij et al., 2018) and GPR143 is able to regulate myocilin release *via* RPE exosomes *in situ* (Locke et al., 2014). Specifically, activation of GPR143 reduces the release of exosomes from the apical side, by recruiting myocilin to the endocytic compartment of ILVs (Locke et al., 2014). While activation of GPR143 caused a reduction in EV release as shown *via* nanoparticle tracking, it would also be interesting to explore the converse – if, in the absence of GPR143, exosomal release (of myocilin) might be increased and whether changes in RPE-derived EV release are linked to pathological changes such as drusen formation (Figure 3B).

Our understanding of the role(s) of RPE-derived EVs in retinal pathophysiology is still very much in its infancy. RPE cells bear all components of the endocytic machinery, are able to phagocytose membranes and release EVs. There is increasing evidence to indicate that EVs are released from the RPE during stress and pathogenic conditions and may even be associated with drusen, but much more work is required to fully elucidate their role in retinal pathophysiology.

### Müller Glia cells release potentially neurotrophic EVs *in vivo*

Müller glia (MG) are the primary glial cell type of the retina providing structural and nutrient support for the neuroretina and regulating the transcellular water transport (Reichenbach et al., 2007; Reichenbach and Bringmann, 2013). Limb’s group, in their latest review, shared preliminary evidence indicating that primary MG cells release small and large EVs, and that these contain the neurotrophins BDNF, NGF and GDNF in either RNA &/or protein form (Eastlake et al., 2021).

Direct evidence of human MG cells releasing EVs has recently been provided by Liu et al., 2022. The targets of these MG-derived EVs and their roles, both beneficial and potentially pathogenic, is only starting to be explored. In the Liu study, administration of MG-derived EVs *in vivo* promotes angiogenesis, potentially *via* a miR9-3p/Sip/AKT/VEGFR2 mechanism (Liu et al., 2022) indicating possible MG-to-endothelial and/or neuron interaction (Figure 3C). MG cells themselves also appear to possess the essential machinery to take up neuronal-derived EVs: we have shown that PR-derived EVs are specifically taken up by MG, and no other retinal cell types, following subretinal and intravitreal administration (Kalargyrou et al., 2021), although the biological relevance of this uptake has yet to be determined. That said, there appears to be some potential for EV-mediated changes in MG function; recently, ESC-derived EVs were administered *in vivo* and reported to induce functional changes in MG including alterations in their morphology and transcriptional profile (Katsman et al., 2012; Gao et al., 2022).

## Extracellular vesicles as potential biomarkers of ocular disease

Despite significant efforts to explore EV biology within the retina and in other tissues, it remains extremely challenging to identify their physiological functions in complex neuronal tissues. It is important to remember that the classical role of EVs is in the disposal of molecular information from cells; as such, they can potentially reflect the cells’ health status and there is considerable interest in their potential as disease biomarkers.

Vesicular structures have been isolated from all eye-related fluids from human subjects including the aqueous humor (Perkumas et al., 2007), the vitreous humor (Ragusa et al., 2015; Zhao et al., 2018) and tears (Grigor’eva et al., 2016) although the latter do not contain neuroretinal-derived EVs. A number of publications to date have focussed on the RNA content of the vesicles isolated from the various ocular fluids. Before considering the potential significance of these findings, it is important to note that many of these studies do not include characterisation of EV cargo related proteins, and ultrastructural analysis is usually missing, limiting our understanding of the types of vesicles present. Moreover, technical details of the methods of RNA isolation are lacking in a number of reports, meaning that care must be taking when considering how well they reflect the transcriptomic profile of the isolated EVs (Grigor’eva et al., 2017; Norman et al., 2021).

The aqueous humor (AH) is the liquid occupying the space between the iris and the cornea (Figure 1). It is presumed that EVs in the aqueous humor are derived from the non-pigmented ciliary epithelial cells, which produce the aqueous humor (AH), although other anterior chamber cells may contribute (Dismuke et al., 2015). Characterization of the AH-derived EVs (AH-EVs) by small RNA sequencing showed an array of miRNA molecules (Dismuke et al., 2015). In order to dissect the cell population responsible for the release, the authors compared the miRNA cargo of AH-EVs with that of EVs produced by primary human trabecular meshwork (HTM) cells (Dismuke et al., 2015). HTM-derived EVs contained a different set of miRNAs but miR-191 and miR-26a were highly enriched in both (Dismuke et al., 2015). This study does not provide any data on EV ultrastructure, or the method of RNA isolation and if the EVs were pre-treated with RNAase and trypsin to eliminate contaminants precipitated from the enrichment method. Therefore, the proposed potential roles of EVs based on their miRNA content (Dismuke et al., 2015; Lerner et al., 2017) remain somewhat speculative and require further exploration.

Nonetheless, the profile of miRNA molecules present in AH fluid (without them necessarily being isolated from AH EVs) has been studied by other groups with non-probe based real time PCR array (Dunmire et al., 2013) and microarray analysis (Tanaka et al., 2014). However, these groups identified different miRNA molecules to those reported by Dismuke and colleagues as being within inside AH-derived EVs (Dismuke et al., 2015). This may be due to the different analysis techniques used but may also reflect that the miRNAs found in AH-derived EVs originate from different cells than those contributing to extracellular miRNAs.

Specific miRNAs have been found in AH derived EVs from uveal melanoma patients (Luz Pessuti et al., 2022), while Gao et al., 2021 provide convincing evidence of the presence of EVs in AH fluids from patients with diabetes and cataracts (DMC), compared with patients with age-related cataracts (ARC) (C. Gao et al., 2021). EM revealed EVs of an appropriate size but the presence of EV tetraspanins was not examined. Differences in the miRNA content of the different samples of AH-derived EVs were reported (C. Gao et al., 2021), but as noted above, the EV samples were not pre-treated with appropriate enzymes (RNAse, trypsin, proteinase K, see Valadi et al., 2007) to eliminate extravesicular contaminants, limiting what can be concluded with regards to biomarker discovery. In another study, Mass Spectrometry analysis of the EV cargo of AH-derived EVs from patients with AMD revealed elevated levels of apolipoprotein A1, C3 convertase, opticin and clusterin proteins, compared to healthy controls (Tsai et al., 2022); the study did not perform RNA anlysis. Although the technical details of the LC–MS are available in this study, further evidence that the protein sample originates from EVs would strengthen their conclusions. The presence of C3 is consistent with the well-reported increases in C3 in the plasma, which are associated with high probability in development of AMD (Tsai et al., 2022); unfortunately, given the challenges in collecting AH fluid compared to blood, this particular change is unlikely to prove useful for diagnostic purposes.

The vitreous humor (VH) is the liquid between the lens and the retina (Figure 1), and it is enriched with EVs (VH-EVs) (Ragusa et al., 2015; Zhao et al., 2018). Given the proximity of the vitreous to the neuroretina, it is expected to contain EVs deriving from neurons, glia and even, potentially, RPE. To our knowledge, no study has yet looked for the presence of cell type-specific markers in VH-EVs and the cells of origin responsible for the release are currently unknown. Nonetheless, there is interest in whether VH-EVs may serve as a potentially useful diagnostic tool; for example, the miRNA profile of vitreous EVs in patients with uveal melanoma (UM), the most common intraocular malignancy in adults, was shown to reflect specific miRNAs being upregulated in the vitreous but not in the patients’ serum (Ragusa et al., 2015; Luz Pessuti et al., 2022). Note that in this study the VH-derived vesicles were characterised with flow cytometry for the presence of tetraspanins, and for their size *via* nanosize tracking analysis. The comparison of VH extracellular miRNAs and the miRNAs contained within VH-EVs confirmed that the miRNA content is different between the UM patients and healthy controls (Ragusa et al., 2015). MiR-21, miR-34a and miR-146a were shown to be increased both in the VH fluid and in the VH-EVs (Ragusa et al., 2015) possibly indicating that melanocytes from UM are releasing EVs. miR-146a was also found to be upregulated in the serum of UM patients compared with healthy donors; expression of miR-146a has been linked with proliferation and tumour formation in mice (Forloni et al., 2014; Ragusa et al., 2015).

Most recently, EVs derived from the vitreous humour from patients with proliferative diabetic retinopathy (PDR) have been reported to exert proangiogenic effects, potentially through the action of miR9-3p (Liu et al., 2022). In this study, VH-EVs were characterised with Western blot analysis for the presence of endocytic markers. Moreover, they were found to contain miR9-3p. Although there is no direct evidence of the cell type producing VH-EVs *in vivo*, the authors assessed RPE-and MG-derived EVs in culture, and concluded MG were the more likely source based on the presence of miR9-3p (Liu et al., 2022). Furthermore, rtQPCR analysis showed that EVs derived from primary cultures of human MG contained miR9-3p when the cells were cultured with high glucose to mimic diabetic conditions, but not when the cells were cultured in low glucose conditions (Liu et al., 2022). Moreover, MG-EVs administered intravitreally were most likely to be up taken by local endothelial cells (Liu et al., 2022). The authors further dissected the downstream signalling pathways, showing that miR9-3p led to the activation of Sip/AKT/ERK cascade (Liu et al., 2022). This study reveals Although direct comparison of the VH-EV cargo seems to resemble that of primary MG-derived EVs, direct proof that the release of miR9-3p from MG cells *in vivo* remains to be shown, although technically challenging. Whilst these results are certainly interesting mechanistically, the vitreous humour is not readily accessible for liquid biopsy and the possible extracted volumes are very small, which will likely limit its utility in terms of routine diagnostics.

## Exploiting extracellular vesicles for retinal therapy

The eye presents a number of anatomical and physiological characteristics that make it an ideal target for the development of novel therapeutic approaches. The anatomical accessibility facilitates surgical interventions, and the retina-blood barrier isolates the eye from other tissues providing it with (at least partial) immune privilege, thereby minimising immunological response to interventions and/or potential side effects in proximal tissues (MacLaren and Pearson, 2007; Buch et al., 2008; Jayakody et al., 2015; Mead et al., 2015; Gasparini et al., 2019; Thomas et al., 2021; Ail et al., 2022). Here, we will discuss the potential for therapeutic administration of EVs in the retina and the combination of gene therapy approaches *via* adeno associated viruses (AAVs) associated with EVs.

### Therapeutic effects of stem cell-derived EVs in the retina

As the complexity and signalling potential of the cargo contained within different EV populations has become apparent, so the interest in their potential as novel therapeutic tools has intensified. The source of EVs (cell of origin), as well as their modification (unmodified EVs derived from cells in culture or modified EVs genetically engineered for a targeted approach), may result in different effects on targeted cells. We will particularly focus on the potential neuroprotective role of unmodified stem cell-derived EVs *in vivo* in the retina.

For EVs to play a therapeutic role, they must be internalised by the target cells. As noted in section 2, there is increasing experimental evidence indicating that RPE, photoreceptors, MG and RPCs are able to release EVs. However, evidence of internalisation is more limited and significant challenges remain to demonstrate the biological significance of EV uptake. The evaluation of EV uptake is important in order to address cell targeting and can generally be achieved in one of two ways: (1) at a cellular level, by utilising fluorescent reporters and visualising the EVs in real time *in vitro* or *in vivo* (Verweij et al., 2018, 2019; Hyenne et al., 2019; Bebelman et al., 2020) or by employing the Cre-loxP system where Cre is delivered *via* EVs into floxed reporter hosts (Zomer et al., 2015; Kalargyrou et al., 2021); (2) at a molecular level, by looking for the presence of EV-specific RNA and/or proteins in the recipient cells (Valadi et al., 2007; Yuan et al., 2009; Katsman et al., 2012; Verweij et al., 2013; Stevanato et al., 2016).

The retina, like the rest of the CNS, lacks any significant regenerative capacity; therefore, loss of retinal neurons due to trauma or disease typically leads to irreversible sight loss. In the case of RGC degeneration, cell therapy strategies utilise stem cells (SCs) in a variety of ways, including transplantation for replacement, but more commonly to provide trophic support to the damaged cells (reviewed in Mead et al., 2015). It has been hypothesised that one of the mechanisms by which SCs mediate their neurotrophic support is *via* the release of EVs. Human bone marrow SC (BMSC)-derived exosomes have been shown to provide greater neurotrophic support to the RGCs compared with fibroblast-derived exosomes during intra-orbital nerve crush (ONC) *in vivo* (Mead and Tomarev, 2017; Mathew et al., 2021). Moreover, microvesicle contamination in the exosome preps inhibits the functional outcome of exosome treatments in culture (Mead and Tomarev, 2017). EVs were administered intravitreally every 7 days post ONC and some functional recovery of RGCs was observed, as measured by electroretinogram (ERG) recordings and assessment of the retinal thickness up to 21 days post-treatment (Mead and Tomarev, 2017). As noted earlier, proving EV internalisation is not trivial. In this study visualisation of the injected EVs involved pre-labelling them with fluorescent membrane dyes, such as DiI. This technique is not particularly robust, however, as the observed DiI signal may be due to droplets formed by the lipid dye itself (van Niel et al., 2021, 2022; Verweij et al., 2021). Nonetheless, the reported functional recovery is promising. To assess whether the exosomal mRNA cargo was responsible for the observed protective effects, the EV-producing BMSCs were transfected with siRNA for Ago2 (siAgo), or scrambled siRNA (siSrc): knocking down Ago2 *via* siAgo reduces the amount of miRNA in EV, compared with scrambled controls (Mead and Tomarev, 2017; Mead et al., 2018). The authors went on to show improvements at a cellular level, showing significantly more GAP43+ RGC axons at the area of injury in the BMSC-exosome treated rats compared to the siAgo BMSC-exosome or fibroblast derived-exosome treated rats, versus untreated controls (Mead and Tomarev, 2017). At a functional level, ERG recordings of the BMSC-exosome treated eyes, showed some improvements of the scotopic ERG amplitudes compared with the untreated controls (Mead and Tomarev, 2017), although these remained reduced compared to uninjured controls. It is worth noting that siAgo BMSC-exosomes treatd eyes also presented with improved ERGs, although the difference was not statistically significant compared to the other controls. This may point towards a neuroprotective role that is not be directly linked to miRNA cargo.

The same group reported similar results using an experimental model of glaucoma, increased intraocular pressure-induced RGC death. Here, intravitreally administered BMSC-exosomes were compared with siAgo BMSC-exosomes, siScr (scramble) BMSC-exosomes and fibroblast derived-exosomes (Mead et al., 2018). Again, scotopic ERG amplitudes were significantly higher in the BMSC-exosome treated eyes, this time with eyes receiving siAgo2 BMSC-exosomes showing little recovery, strengthening the hypothesis that EV-mediated delivery of miRNAs is involved in the rescue (Mead and Tomarev, 2017; Mead et al., 2018). After analysing the miRNA content of the BMSC-exosomes with RNAseq, the authors found three miRNAs to be enriched, miR-144-5P, miR-126-5P and miR-100-5P (Mead et al., 2018). The same group have recently shown that delivery of specific combinations of miRNAs (miR-17-5p/miR-30c-2/miR-92a or miR-92a/miR-292/miR-182) to the retina *via* Adeno-associated virus 2 (AAV2) exerts neuroprotective effects in ONC injury (Mead et al., 2020). Whilst the efficiency of delivery of miRNAs *via* AAV-mediated expression is substantially higher than could be expected *via* EV-mediated transfer, it is notable that these miRNA molecules were also found in the cargo of BMSC-derived EV preparations (Mead et al., 2020).

To examine the bioavailability of BMSC-derived EVs in the retina, Roth’s group followed the distribution of fluorescently labelled BMSC-derived EVs using daily fundoscopy for 7 days after a single intravitreal injection (Mathew et al., 2021). Fundoscopy lacks sufficient resolution to make any meaningful assessments regarding the cellular distribution of the labelled EVs, but GFP fluorescence declined rapidly. The authors propose that this may indicate rapid clearance, by microglia or astrocytes (Mathew et al., 2021). This is in keeping with recent evidence in zebrafish where the majority of EVs released in the bloodstream are cleared by macrophages (Hyenne et al., 2019; Verweij et al., 2019). However, such reductions might also be expected if the EVs are internalised and degraded by the recipient cells. If exogenous EVs are indeed rapidly cleared from the eye by the immune system, it raises important considerations about their therapeutic utility, if they need to be administered *via* repeated intraocular injections.

Surprisingly, human Mesenchymal Stem Cell (hMSC)-derived EVs were reported to exert neuroprotective effects during ONC when administered *via* the tail vein (Seyedrazizadeh et al., 2020). Specifically, either MSCs, or MSC-EVs or MSC-conditioned media were injected every two days for 6 days from the day of ONC injury, and the eyes were analysed at 21-and 60-days post administration. Although injected into the bloodstream, 30 min after administration, calcein fluorescence was detected in the eye, which the authors propose showed the presence of MSC-EVs in the vitreous. Although EV visualisation was not compelling, MSC-EVs appear to support a modest improvement in RGC survival, as assessed *via* quantification of the number of RGC axons per field of view in retinal flat mounts and by measurements of nerve fibre layer thickness (Seyedrazizadeh et al., 2020). However, the effect was not sustained, and any potential neuroprotective effects were lost by day 60 (Seyedrazizadeh et al., 2020). It is perhaps surprising therefore that the authors observed improvements in a behavioural test of visual function, the visual cliff test, at day 60 in those animals receiving either MSCs or MSC-EVs, compared with ONC-only controls (Seyedrazizadeh et al., 2020). No assessments of retinal function (e.g., ERGs) were made. It is not yet known what factors within the MSC-EVs (and cells) supports RGC survival, at least in the short term, although others have indicated that MSC-derived EVs may mediate neuroprotective effects by modulating the PI3K/Akt pathway (Cui et al., 2021).

An interesting recent approach has been to engineer EVs to enable binding of specific cargo proteins in order to achieve their targeted delivery. In 2018, Gao et al., demonstrated that by utilising the anchor peptide CP05, which binds to the extracellular region of tetraspanin CD63, they were able to bind specific cargo and achieve targeted delivery by EV uptake (Gao et al., 2018). Most recently, they utilised this approach to deliver pituitary adenylate cyclase-activating peptide (PACAP38) to injured RGCs (Wang et al., 2021). PACAP is a neuropeptide that specifically binds the G-coupled protein receptors (GPCRs) VPAC_1_, VPAC_2_ and PAC_1_, and exerts a neuroprotective function (Rubio-Beltrán et al., 2018). To achieve enhanced and targeted delivery of PACAP38, the authors modified EVs produced by RGCs to carry PACAP38 and deliver it to other RGCs in the expectation of achieving neuroprotection (Wang et al., 2021). PACAP38-positive RGC-EVs were administered intravitreally to animals undergoing optic nerve crush injury (ONC) and the authors observed increased RGC survival and axonal regeneration *in vivo*. These findings are undoubtedly interesting, but also raise a number of questions from a mechanistic perspective. A key one is how PACAP38 is delivered once the EVs are internalised: Is PACAP38 cleaved from the EV membrane, or is the expectation that the lipid moieties (and associated PACAP38) of EVs are incorporated into target cell membrane? Did the anchoring of PACAP38 at C05 peptide introduced a more potent form of PACAP38? If so, could liposomes be similarly functionalised, to avoid the laborious process of generating and isolating EVs from RGCs, or are other features of the EV critical for uptake? Such considerations are pertinent to the broader use of EVs as vectors for targeted delivery of proteins.

In addition to neuroprotective actions, pro-regenerative roles have been proposed for hESC-derived EVs. These EVs have been shown to contain BDNF and appear to induce at least partial de-differentiation of MG cells *in vitro*, as indicated by immunostaining for Pax6, SOX2 and PH3, as well as BrdU incorporation (Gao et al., 2022). However, further work is required to establish if this reflects a true de-differentiation with the ability to generate new neurons *in vivo*. As we have discussed elsewhere (Pearson and Ali, 2018) lineage tracing techniques will be crucial to validating such a hypothesis.

### Harnessing EVs for gene therapy

Gene delivery is typically achieved *via* the use of two types of vectors; (1) viral vectors, such as recombinant adeno-associated viruses (rAAV) and lentiviruses, and (2) non-viral vectors, such as nanoparticles (e.g., liposomes) and most recently, EVs. Important considerations when deciding on the appropriate viral vector for efficient gene delivery are the packaging capacity, tropism (different serotypes preferentially transduce certain cell types over others) and the immunogenicity (presence of neutralising serum antibodies). Further specificity can be introduced with cell-type specific promoters upstream of the transgene. The rAAVs exhibit several advantages for retinal gene therapy, being non-pathogenic, single-stranded DNA viruses that efficiently transduce a wide variety of retinal cell types, including photoreceptors and RPE cells, and can mediate stable expression in the retina for several years (Bainbridge et al., 2003). The tropism of various adeno-associated virus type 2 (AAV2) pseudotypes has been extensively studied in the eye *in vivo* in mice (Watanabe et al., 2013; Kleine Holthaus et al., 2018) and primates (Hickey et al., 2017), in organotypic human retinal cultures (Wiley et al., 2018) and in human retinal organoids (Gonzalez-Cordero et al., 2018; McClements et al., 2022).

Interestingly, AAV vectors are able to directly associate with EVs (microvesicles and exosomes), either within the vesicle’s lumen or on their membrane (Maguire et al., 2012; Fitzpatrick et al., 2014; György et al., 2014, 2017; Wassmer et al., 2017). Due to the clinical applications of AAVs for corrective gene delivery, it is worth noting that EV-associated AAV vectors exo-AAV1 and exo-AAV2 were highly resistant over a range of neutralizing antibodies from human serum, compared with standard AAV vectors *in vitro* and *in vivo*, most likely due to the inaccessibility of AAV antigens inside the EV lumen (György et al., 2014). Following these results, the same group compared the infectious profile of conventional AAV2 and exo-AAV2-FLuc vectors in the retina *via* intravitreal administration and subsequent tracking of GFP expression levels with fundoscopy and immunohistochemistry (Wassmer et al., 2017). Transduction of the RGC layer by exo-AAV2 vectors, compared with conventional AAV2 vector, was enhanced, as was transduction of the INL.

The mechanisms behind the more efficient gene transfer achieved by the exo-AAV compared with AAVs have yet to be determined. AAVs do not necessarily share the same routes of entry in the cells with EVs regarding their uptake, as EVs do not appear to have the same fusogenic properties with viruses (van Dongen et al., 2016). However, AAV2 can bind to the cell surface specifically *via* Heparan Sulphate Proteoglycans (HSPGs) (Summerford and Samulski, 1998) and a similar mechanism has been shown to be involved in the uptake of EVs (Christianson et al., 2013; Christianson and Belting, 2014). Another potential mechanism facilitating the observed improvements in transduction maybe the lipid moieties of the EVs, specifically cholesterol and phosphatidylserine (reviewed in Skotland et al., 2019) facilitating internalisation (Segawa and Nagata, 2015) or it may be a matter of EVs concentrating more AAV particles within their lumen and membrane, making delivery more efficient (Hudry et al., 2016).

Following these results, exo-AAV2/9 has been reported to even cross the blood brain barrier and transduce neuronal cells *in vivo* and, interestingly, maintain its typical tropism profile in the CNS (Hudry et al., 2016). Moreover, exo-AAV2/9 outperforms traditional AAV2/9 with respect to transducing both neurons and glia when administered systemically (Hudry et al., 2016); a key next step will be to determine whether exo-AAV2/9 administered systemically can equate to or even outperform AAV2/9 injected directly into the CNS since this would circumvent the need for intracranial surgery and could be a major advance from a clinical perspective.

The inner ear shares some similarities with the retina with regards to its lamination, and, in keeping with the findings in the retina, exoAAV1 and exoAAV9 serotypes both showed better efficiency of transduction - as assessed *via* GFP fluorescence - in the inner hair and cochleal cells in explant cultures and *in vivo*, compared with the conventional AAV1 and AAV9 vectors (György et al., 2017). By employing exoAAV1 to deliver the murine *Lhfpl5* gene *via* cochleostomy and Round Window Membrane (RWM) injections in the deaf *Lhfpl5/Tmhs^−/−^* mouse, the authors reported partial recovery of hearing (György et al., 2017). The functional recovery was assessed at a cellular level with auditory brainstem response, by different tone stimuli and at a behavioural level with startle response (György et al., 2017). The reported rescue of hearing *via* utilising the exoAAV1 approach in the inner ear opens a new area of study towards more efficient gene delivery that may be exploited clinically in the retina.

In summary, exoAAVs have been shown to achieve more efficient delivery of viral DNA and/or RNA molecules compared with conventional AAVs in neuronal and glial cultures (Kovács et al., 2021), in the brain (György et al., 2014), retina (Wassmer et al., 2017), and auditory sensory system (György et al., 2017).

## Discussion

The past decade has seen enormous interest in EVs and their potential role in physiology, pathophysiology and therapy, with many papers attributing EVs with a role in long range cell–cell communication. Numerous studies do indeed describe the release and potential uptake of a variety of vesicles from many different cell types and the retina is no exception. EVs have been attributed with functions around retinal development and differentiation, signalling during conditions of stress and injury, and providing neurotrophic support. These potential roles are very exciting; however, in many cases, experimental EVs have been produced outside of the organism, using cell culture, and administered exogenously and sometimes they even originate from different species (see section 4.1). Therefore, care is needed when trying to extrapolate to potential signalling roles in the physiology of the healthy tissue. On the other hand, EVs, independent of their biogenesis or species origin, can be internalised and can affect a variety of cell functions. In this review we have explored some of the potential roles and applications of EVs in the retina.

We begin with the axiom that all cells are able to release EVs (Raposo and Stoorvogel, 2013); whilst it is true that several types of retinal cells can release a plethora of vesicles in culture, be that 2D cell culture, organoids or organotypic tissue cultures, it is less clear if they do so under physiological conditions *in vivo*. Indeed, the answer likely depends on the cell type and stage of development in consideration. Evidence to date suggests that RPE cells are likely to release EVs in the retina (Locke et al., 2014; Klingeborn et al., 2017; Shah et al., 2018; Flores-Bellver et al., 2021) and such release mostly reflects cell functions around phagocytosis/degradation. We have shown that early postnatal photoreceptors can release EVs *in vitro* and the same cells bear the machinery required to make and release EVs *in vivo* during postnatal development (Kalargyrou et al., 2021), but we have yet to establish whether such release actually occurs. Similarly, it would seem plausible that MG release EVs *in vivo*, much as they do *in vitro* (Eastlake et al., 2021), and there is good evidence to suggest that they are capable of taking up EVs in the intact retina (Kalargyrou et al., 2021). What role, if any, does such uptake play in intercellular communication in the retina? Given their known roles in the degradation pathway, it seems most likely that they constitute a damage response in retinal dysfunction. Do degenerating photoreceptors release EVs as a stress signal? These could be detected by Muller glia, RPE or local immune cells. To begin to unpick the possibilities, it will be necessary (although undoubtedly technically challenging) to establish EV release in the intact retina under normal and pathological conditions, utilising live imaging techniques similar to those used elsewhere (Zomer et al., 2015; Hyenne et al., 2019). The tetraspanin pHluorin system of EV visualisation (Verweij et al., 2019) might be combined with specific cell promoters to permit identification of those cells releasing EVs as well as the fate of those EVs.

EVs have been identified in both the aqueous and vitreous humor and there is much interest in their potential utility as biomarkers for disease. Given the proximity of the vitreous to the neuroretina, it is expected that this chamber will contain EVs deriving from neurons, glia and, potentially, RPE. However, to our knowledge, no study has looked for the presence of cell-specific markers in the vitreous-EVs, which limits our ability to interpret the information obtained from these EV populations. The accessibility of vitreous humor for liquid biopsy and the size of the extracted volumes poses further challenges. One should also consider the technical limitations of the dealing with small sample volumes for appropriate purification and subsequent EV cargo analysis: Biomarker discovery in the brain has shown that several molecules thought to be enriched in EVs, such as L1CAM, may be present as contaminants due to sample preparations, rather than residing in the lumen of EVs derived from CSF and plasma of patients with neurodegeneration (Norman et al., 2021). Moreover, purified EVs of any origin should be devoid of contaminants such as serum proteins and proteins of intracellular compartments (e.g., the endoplasmic reticulum or mitochondria) (Raposo and Stoorvogel, 2013). Unfortunately, few studies of vitreous and aqueous derived EVs provide a comprehensive characterisation of the isolated vesicles; while this may not prevent results from such analysis being useful in terms of biomarker changes, it makes it harder to attribute a function to EVs in the process. Indeed, the lack of characterisation of EV samples in many publications seeking to use them as biomarkers or indeed apply them as therapeutic agents needs to be addressed ahead of any future clinical applications.

There has been extraordinary interest in the potential of EVs as therapeutic agents (summarised in Lener et al., 2015). In the retina, administration of native or modified EVs, either *in vitro*, *ex vivo* or *in vivo,* has been linked to an array of effects in recipient cells, with improvement in RGC survival after ONC being one of the more promising models explored (Mead et al., 2015, 2016, 2018, 2020; Mead and Tomarev, 2017). However, our understanding of the mechanisms behind such neurotrophic effects, and precisely what within EVs mediates the effects, is still limited. The immunogenicity of EVs, as well as potential toxicity effects (Zhu et al., 2017) should also be carefully assessed, together with considerations around the route of administration, prior to clinical applications. The use of EVs in gene therapy (exoAAV) may hold promise, particularly if it can be convincingly shown that exoAAVs introduced *via* systemic administration can outperform direct administration of normal AAVs, potentially circumventing the need for injections into the brain.

There is much to excite about the potential of EVs for retinal research, from intercellular signalling in development ahead of synapse formation to cell stress signals in injury and disease. Diversity of cell origin, and biogenesis, together with defining the cargo content of EVs, are the biggest challenges for accurately defining their role in normal and pathological retinal physiology. Also critical is the need for robust evidence of EV release in the intact eye, be it healthy and/or diseased. Greater attention should also be given to the lipid composition of retinal EVs along with the molecular cargo, as lipid moieties may serve as signals for internalisation. Each of these challenges will become easier, however, as the field of EV research is exacting increasing rigor with the publication of multiple position papers (Hill et al., 2013; Lener et al., 2015; Théry et al., 2018) and guidelines that are under constant evaluation. Similarly, the interest in the field is driving the development of newer and better visualisation techniques.

## Author contributions

AK and RP: (CRediT taxonomy) conceptualization. AK: writing--first draft. AK, AS, and RP: review and editing. SG and AK: drawing. RA and RP: funding acquisition and supervision. All authors contributed to the article and approved the submitted version.

## Funding

This study was conducted in Kings College London. This work was supported by Medical Research Council UK (MR/T002735/1).

## Conflicts of interest

The authors declare that the research was conducted in the absence of any commercial or financial relationships that could be construed as a potential conflict of interest.

## Publisher’s note

All claims expressed in this article are solely those of the authors and do not necessarily represent those of their affiliated organizations, or those of the publisher, the editors and the reviewers. Any product that may be evaluated in this article, or claim that may be made by its manufacturer, is not guaranteed or endorsed by the publisher.
